# Cross-sectional E-survey on the Incidence of Pre- and Postoperative Chronic Pain in Bariatric Surgery

**DOI:** 10.1007/s11695-022-06354-9

**Published:** 2022-11-08

**Authors:** Bart Torensma, Mohammed Hany, Marije J. S. Bakker, Monique van Velzen, Bas A. in ’t Veld, Albert Dahan, Dingeman J. Swank

**Affiliations:** 1 Department of Anaesthesiology, Leiden University Medical Center (LUMC), Albinusdreef 2, 2333 ZA Leiden, the Netherlands; 2 Medical Research Institute, Alexandria University, Alexandria, Egypt; 3 Dutch Obesity Clinic West, the Hauge, the Netherlands; 4 Haaglanden Medical Center, the Hauge, the Netherlands

**Keywords:** Survey, Incidence, Chronic pain, Chronic abdominal pain, Bariatric surgery, Pre- and postoperative

## Abstract

**Background:**

To assess the prevalence, incidence, location, and behavior of chronic pre- and postoperative pain in bariatric surgery, and the use of analgesics.

**Methods:**

A cross-sectional e-survey was conducted on 3928 post-bariatric patients and four-time points for pain assessment were evaluated: preoperative, on the ward, day 1 at home postoperatively, and present time (at the time of the e-survey). A numerical rating scale (NRS) was used to assess the level of pain (0 to 10). The general incidence of chronic pain was calculated, as also, subgroups were defined as group A (pre and postoperative chronic pain), B (preoperative pain, and no longer postoperative), and C (preoperative painless, postoperative chronic pain). Besides the pain intensity, location of pain, and the use of analgesics were investigated.

**Results:**

A total of 3279 patients (83.9%) responded to the survey. Preoperative and postoperative chronic pain was found in 343 (10.5%) and 264 (8.1%) patients, respectively. In group A, chronic pain was present in 4.8% of the patients; in group B, it was present in 5.7%; and in group C in 3.3% of the patients. Furthermore, in 4.5% of patients pain was located in the abdomen, which was higher as compared to before surgery (+ 2.3%, *p* < 0.001). The ORs for present postoperative chronic pain were OR 1.45, 1.7, and 1.71 (*p* = 0.002, 0.003, 0.003) compared to respectively preoperative chronic pain, pain at the ward, and pain at day 1 after surgery. Among all participants, 4.6% consumed chronic analgesics. Of these, paracetamol was used most frequently (3.8%), followed by tramadol (1.3%) and oxycodone (0.5%).

**Conclusions:**

In this e-survey, chronic postoperative abdominal pain was prominent in patients after bariatric surgery. Of patients, 3.3% that were without preoperative chronic pain developed chronic pain after surgery. Opioid consumption in the queried population was relatively low.

**Graphical Abstract:**

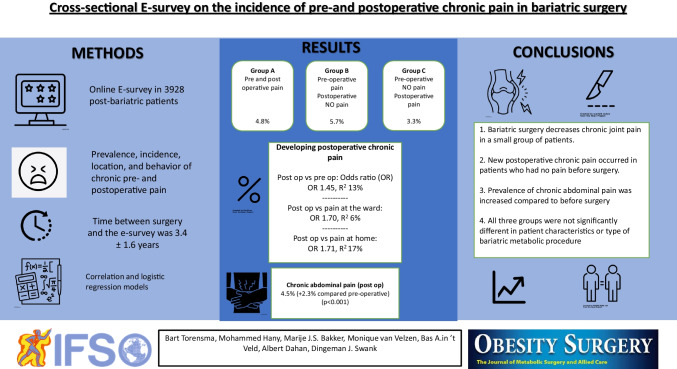

**Supplementary Information:**

The online version contains supplementary material available at 10.1007/s11695-022-06354-9.

## Introduction

The World Health Organization has officially declared obesity as a global epidemic. Currently, more than 650 million (13%) adults suffer from obesity, which is defined as a body mass index (BMI) of 30 kg/m^2^ or greater [1]. Obesity at an early age can influence pain perception later in life [2, 3].

Acute post-surgical pain occurs in the first 1 to 3 months after surgery. The duration and intensity of post-surgical pain varies with the type and duration of surgery, patient factors such as gender, race, age, and surgical complications [4, 5]. In 2% to 14% of surgical patients, pain following surgery persists for more than 3 months and is known as chronic or persistent postoperative pain [6]. Interestingly, several studies report the incidence of chronic pain after bariatric metabolic surgery (BMS), especially abdominal pain, in up to 61.4% of patients [7–14]. Adequate treatment of acute postoperative pain can reduce the risk of persistent postoperative pain.

Postoperative pain is commonly treated according to a multimodal approach that includes paracetamol, non-steroidal anti-inflammatory drugs, and opioids. Opioids bind in plasma to the transporter protein alpha-1-acid glycoprotein. The concentration of this protein is usually higher in patients with a chronic inflammatory state such as obesity. As a consequence, the free fraction of the opioid is expected to be lower and as a consequence a reduced analgesic effect of opioids; patients with obesity likely need higher dosages to achieve similar effects [6].

For a better understanding of perioperative pain in patients undergoing obesity surgery, knowledge of the prevalence, type of pain, and duration of pain, research is necessary. To our knowledge, this is not yet investigated/studied. Therefore, in this study, we conducted a cross-sectional e-survey for the prevalence and location of chronic pre- and postoperative pain, the incidence of new or disappeared chronic postoperative pain in bariatric surgery, and medication use.

## Methods

### Study Design

A cross-sectional e-survey was conducted between August 2017 and January 2019 at the Dutch Obesity Clinic west, The Hague, the Netherlands. To assess the prevalence and incidence of chronic pre- and postoperative pain in patients undergoing bariatric surgery. Written consent was obtained from all the patients. The data were analyzed anonymously. The study was conducted in accordance with the principles of the Declaration of Helsinki and was approved by the local ethical committee of the Dutch Obesity Clinic medical research institute before the e-survey was sent.

### Study Endpoints

The prevalence and location of chronic pre- and postoperative pain, the incidence of new chronic postoperative pain, and the use of medication after bariatric surgery.

### Inclusion Selection

All patients in this survey met the inclusion criteria prior to surgery. Therefore, all the patients were preoperatively screened and indicated for bariatric surgery according to the International Federation for the Surgery of Obesity and Metabolic Disorders (IFSO) criteria. They were selected from hospital electronic databases on primary sleeve gastrectomy or RYGB procedures and contacted regarding participation in this survey. All patients who denied participation were excluded from the study.

## Data Collection

The questionnaire was sent at least three months after the surgery was performed. The baseline characteristics of the patients included pre- and postoperative body mass index (BMI); the weight was measured before surgery at the clinic and after surgery by the patient itself.

The pain was assessed at four different time points: preoperative, at the ward, 1 day after surgery at home, and at the time of the e-survey assessment. A numerical rating scale (NRS) was used to assess pain intensity on a scale of 0 to 10 with zero indicating no pain and 10 being the most pain imaginable. Besides the pain localization, the intake of analgesics was also evaluated.

### Chronic Pain

Chronic pain was defined as pain during at least three months intermittently present in daily life. Besides the general incidence of chronic pain, this study also defined three subgroups of patients.

- A: Patients who had preoperative pain and continuing postoperative
- B: Patients who had preoperative chronic pain but are no longer postoperative.
- C: Patients without preoperative chronic pain but suffering from chronic pain postoperative.

### Data Capture

The analysis was performed on a blinded data set after the medical/scientific review was completed and all protocol violations were identified and solved, and the data set was declared complete. All data and survey questionnaires (Appendix 1) were collected in a data management system (Castor EDC, Amsterdam, the Netherlands; https://www.castoredc.com) and handled according to Good Clinical Practice guidelines, Data Protection Directive certificate, and complies with Title 21 CFR Part 11. Furthermore, the data center where all the research data was stored was ISO27001, ISO9001 certified, and Dutch NEN7510 certified.

### Statistical Methods

We used descriptive statistics and inferential statistics. All data were first tested for normality by a Kolmogorov–Smirnov test, a *Q*-*Q* plot, and Levene’s test. Categorical variables were expressed as *n* (%). Continuous normally distributed variables were represented by their mean and standard deviation, discrete by their median and interquartile range for skewed distributions. To compare categorical variables among different groups, the Pearson’s Chi-square test or Fisher’s exact test was used, when appropriate, and in case of paired groups by the McNemar test. Normally distributed continuous data were tested with the independent samples, and Student’s *t*-test was used to compare continuous variables between independent samples and, in the case of skewed data, the Mann–Whitney *U*-test was used. The correlation between the NRS scores in different phases was determined using a Pearson product-moment correlation coefficient for normally distributed data or Spearman’s rank correlation coefficient for non-normally distributed data. Logistic regression was used to determine the odds ratio of developing chronic pain. *P-*values of < 0.05 were considered statistically significant. Statistical analyses were performed using R (version 4.0.4) packages.

## Results

### Baseline Characteristics

A total of 3928 patients were approached between August 2017 and January 2019. From these, 83.9% responded with informed consent to participate in the study (*n* = 3279). Sleeve gastrectomy (LSG) and Roux-en-Y gastric bypass (RYGB) surgeries were performed in 16.9% and 83.1% of patients, respectively. Age at the time of the survey was 49 ± 11 years. In our cohort, 82.5% of patients were female and 17.5% were male. The mean time between surgery and the e-survey was 3.4 ± 1.6 years. Surgery periods were between 2009 and 2018.

Preoperative weight and weight at the survey were 129 ± 22 kg and 87 ± 19 kg, respectively, with an average weight loss of 42 ± 16 kg. The pre- and postoperative BMI was 45 ± 6 and 30 ± 6 kg/m^2^, respectively. The percentage excess weight loss (%EWL) was 68.3 ± 24.7%,and % total weight loss (%TWL) was 31.8 ± 10.4 (Table 1).

**Table 1:** Demographics and pre- and postoperative incidence of chronic pain

Total *n* = 3279	Demographics		
Gender			
Male *n* (%)	574 (17.5)		
Female *n* (%)	2705 (82.5)		
Age at e-survey mean ± sd	49.16 ± 11.2		
RYGB^§^	2724 (83.1)		
Sleeve gastrectomy	555 (16.9)		
Time between operation and e-survey—years ± sd	3.43 ± 1.61		
	Preoperative	Postoperative	*p* value^*^
Weight (kg) mean ± sd	129.10 ± 21.63	87.37 ± 18.73	< 0.001^a^
BMI (kg/m^2^)	44.7 ± 6.0	30.3 ± 5.7	< 0.001^a^
%EWL	-	68.04 ± 24.05	-
%TWL		31.8 ± 10.4	-
Incidence of chronic pain *n* (%)			
Incidence of chronic pain	343 (10.5)	264 (8.1)	< 0.001^b^
Patients who had preoperative pain and continuing postoperative	156 (4.8)	-	-
Patients who had preoperative chronic pain but are no longer postoperative	186 (5.7)	-	-
Patients without preoperative chronic pain but suffering from chronic pain postoperative	-	108 (3.3)	-
Location of pain *n* (%)			
Back	213 (6.5)	149 (3.8)	< 0.001 ^b^
Pelvis/buttocks/thighs	121 (3.7)	75 (2.3)	< 0.001 ^b^
Knee	213 (6.5)	72 (2.2)	< 0.001 ^b^
Lower legs/feet	151 (4.6)	54 (1.6)	< 0.001 ^b^
Abdomen	73 (2.2)	149 (4.5)	< 0.001 ^b^

^*^p < 0.05 was considered statistically significant

^a^Paired sample *T*-test

^b^McNemar paired test

^§^Roux-en-Y gastric bypass

### Incidence of Preoperative Chronic Pain

Preoperative chronic pain was noted in 10.5% (*n* = 343) of patients with a NRS of 6.9 ± 1.6. Preoperatively, the pain occurred in the back, knee, pelvis/buttocks/thighs, lower legs/feet, and abdomen in 213 (6.5%), 213 (6.5%), 121 (3.7%), 151 (4.6%), 73 (2.2%) patients, respectively (Table 1).

There were no significant differences between LSG and RYGB procedure selection and the incidence of preoperative chronic pain (*p* = 0.118).

### Incidence of Pain at the Ward

After surgery on the ward, 799 (24.4%) of patients experienced acute pain with a NRS of 6.6 ± 1.86. There were no significant differences between LSG and RYGB procedures in the presence of pain at the ward (*p* = 0.067), but the patients with an LSG procedure had significantly higher NRS scores (NRS 7.18 ± 1.97 vs. 6.57 ± 1.82) (*p* = 0.0004).

### Incidence of Pain at Home

Postoperative, 1 day after surgery, 779 (23.8%) patients experienced acute pain with a NRS of 5.7 ± 1.9. There were no significant differences between LSG and RYGB procedures in the presence of pain at home (*p* = 0.148) or NRS scores (*p* = 0.776).

### Incidence of Postoperative Chronic Pain (Compared to Preoperative)

Postoperative pain had an incidence of 8.1% (*n* = 264) with a NRS of 5.9 ± 1.9.

Pain postoperative was located at the back (n = 149, 3.8% (− 2,7% compared to preoperative) (*p* < 0.001)), pelvis/buttocks/thighs (*n* = 75, 2.3% (− 1.4%) (p < 0.001)), knee (*n* = 72, 2.2% (− 4.3% (*p* < 0.001)), lower legs/feet (*n* = 54, 1.6% (− 3.0%) (*p* < 0.001)), and abdomen (*n* = 149, 4.5% (+ 2.3% (*p* < 0.001)), respectively (Table 1).

There were no significant differences between LSG and RYGB procedures in the presence of postoperative chronic pain (*p* = 0.431).

### Sub-group Analysis of Chronic Pain Postoperative

- Group A: pain was present in 4.8% of patients. (156 out of 3279 patients)
- Group B; pain was present in 5.7% of patients. (186 out of 3279 patients)
- Group C; pain was present in 3.3% of patients. (108 out of 3279 patients) (Table 1)

All three groups were not significantly different in patient characteristics (*p* = 0.873).

### Correlation of Developing Postoperative Chronic Pain

The odds for the development of postoperative chronic pain as compared to preoperative chronic pain was OR 1.45 (CI95% 1.2–1.69) (*p* = 0.002), with a significant coefficient of determination (COD) on the NRS score of *R*^2^ 13% (*p* = 0.003).

The odds for the development of postoperative chronic pain compared to the presence of pain at the ward was OR 1.70 (CI95% 1.46–1.94) (*p* = 0.003), with a significant COD on the NRS score of *R*^2^ 6% (*p* = 0.001).

Finally, the odds for the development of postoperative chronic pain as compared to the presence of pain at home was OR 1.71 (CI95% 1.47–1.95) (*p* = 0.0048), with a significant COD on the NRS score of *R*^2^ 17% (*p* = 0.0024).

### The Use of Analgesics

Overall, 4.6% of all respondents were on chronic analgesics. Among these, paracetamol was the most used drugs (3.8%), followed by tramadol (1.3%), oxycodone (0.5%), diclofenac (0.4%), naproxen (0.3%), ibuprofen (0.2%), amitriptyline (0.2%), paroxetine (0.2%), citalopram (0.2%), diazepam (0.2%), morphine (0.1%), fentanyl (0.1%), and pregabalin (0.1%).

## Discussion

In this study, a cross-sectional e-survey was performed in 3279 patients after bariatric surgery. The preoperative prevalence and the postoperative incidence of pain were 10.5% and 8.1%, respectively. Sub-group analysis of chronic pain revealed that, in group A, pain persisted in 4.8% of patients, in group B (5.7%), pain was present preoperative but no longer after the bariatric operation, and in group C (3.3%), new pain started after the operation.

Chronic postoperative pain is associated with decreased quality of life, sleep and mood disorders, and neuropathic symptoms [5]. Additionally, chronic pain is associated with increasing BMI, and can be caused by a combination of lifestyle and mechanical, chemical, and psychological mediators [15, 16]. Postoperative chronic abdominal pain has been reported in 30% of patients after a Roux-en Y gastric bypass [7, 9, 10, 17–20]. Besides this, a range of other types of pain is also noted in patients undergoing bariatric surgery. To our knowledge, this is the first study that investigated multiple aspects of pain in a large cohort of bariatric patients.

In our study, after bariatric surgery, BMI decreased over time (44 to 30 kg/m^2^, EWL 68.3%) leading to a reduction in chronic pain by 5.7% (group B), with predominantly a reduction of musculoskeletal pain in the back, pelvis, knees, and lower legs/feet. This was not unexpected as bariatric surgery–induced weight loss has been earlier shown to significantly improve musculoskeletal pain [21]. For example, one study showed that joint pain decreased from 71 to 57% after 5 years of bariatric surgery [8]. Similarly, in our study, 52 to 67% reduction in joint pain was observed after bariatric surgery and weight loss. Furthermore, we detected a new group of patients with chronic postoperative pain (3.3%, group C) who had no history of pain prior to surgery. Previous studies mostly evaluated the overall incidence of postoperative pain; however, to our knowledge, none of them focused on patients who were pain-free before surgery.

Our study found an increased OR in the development of postoperative chronic pain; this highlights the importance and attention to pain treatment during the preoperative screening, hospital admission, and discharge phase. First, our study found that when preoperative chronic pain is present, this is correlated with postoperative chronic pain (OR of 1.45 and *R*^2^ of 13%). In this study, these patients experienced chronic pain preoperatively and retained this pain after the operation (group A). Group B in this study also had preoperative chronic pain, but this pain was not present anymore after surgery. There were no significant differences between the patient's characteristics between groups A and B, so therefore, predicting what patients will have postoperative (chronic) pain remains unanswered, but this is an important field for future research.

Furthermore, this study showed that in patients who presented with pain at home postoperatively, this pain was correlated by 17% in the development of chronic pain (OR 1.71). Therefore, it is important that before discharge, proper pain medication and instructions for the follow-up phase are in place.

Furthermore, 2.2% of the patients in our survey had preoperative chronic abdominal pain. We observed a twofold increase in abdominal pain postoperatively, which is in accordance with previous reports [7, 9, 10, 17–20]. Abdominal pain has been identified as the most common postoperative symptom (61.4%) in patients leading to hospital readmissions [7, 8, 8–14] and reduced health-related quality of life [7]. Therefore, there is a need to conduct longitudinal evaluations before and after bariatric surgery and establish relationships between BMS, patients with obesity, and the development of chronic (abdominal) pain.

There are several speculations regarding the etiology of chronic abdominal pain after bariatric surgery: (1) behavioral and dietary disorders such as over and rapid eating resulting in mechanical distension of the pouch or reduced stomach; (2) functional disorders such as constipation, diarrhea, irritable bowel syndrome, and dumping syndrome; (3) biliary disorders such as cholelithiasis, choledocholithiasis, or sphincter of Oddi dysfunction; (4) pouch or remaining stomach disorders such as peptic ulcer disease, gastro-gastric fistula, gastroesophageal reflux disease (GERD), hiatus hernia, and internal hernias; and finally (5) disorders in the abdominal wall like anterior cutaneous nerve entrapment syndrome (ACNES) [13, 17, 22–24]. However, the pain etiology remains unknown in up to half of all cases with abdominal pain after RYGB, and the association with other forms of bariatric surgery is even less evident [12, 13, 23].

The US Center for Disease Control reported an increase in opioid use in patients with persistent pain after bariatric surgery [25]. In a survey study from the USA, it was identified that before surgery, 8% (*n* = 933) of bariatric patients were chronic opioid users. Of this group, 77% continued using chronic opioids the year after surgery. On average daily use of 45.0 mg morphine equivalents preoperative and 51.9 mg daily postoperative (*p* < 0.001), there was an increase in opioid consumption after the bariatric surgery. Furthermore, the change in morphine equivalents before versus after surgery did not differ between individuals who lost more than 50% excess weight loss vs. those with 50% or less [26].

Another study testing the effect of new persistent opioid use on physiologic and psychologic outcomes following bariatric surgery found a new persistent opioid use of 6.3% (out of 27,799 patients). In this study, the excess body weight after 1 year of bariatric surgery was less, compared to the group without new persistent use. Also, new persistent opioid use had significantly worse psychological well-being, body image, and depression score [27]. The Swedish nationwide cohort study on 56,183 patients after bariatric surgery reported that 17.5% of the 56,183 patients had been on at least one opioid prior to surgery, and 4.3% of these patients were defined as chronic opioid users. That same study showed in active opioid users, a higher risk of severe complications (OR 1.67), increased length of hospital stays (OR 1.11), and higher rates of readmissions and reoperations (OR 1.70 and 1.87, respectively) after bariatric surgery [28].

In contrast to the US data, we detected that chronic analgesics were consumed by a low proportion of patients (4.6% of all respondents), with opioid consumption much lower than reported in US studies [26, 27]. Irrespective of location (the USA or the Netherlands), we argue that postoperative pain, in combination with chronic (opioid) use, requires specific attention in the pre- and postoperative phase so that chronic opioid use can be prevented.

This study showed that treatment with bariatric metabolic surgery has positive and negative effects on the presence of pain in a patient with obesity. Our study presented an overview of the incidence and correlations of pain, but questions remain unanswered on the characteristics of the patients prone to develop chronic pain.

The call to action should focus on research on the causes and effects of pain in patients with obesity in the pre-peri- and postoperative phases of surgery.

### Limitations

This e-survey also had some limitations. We performed a retrospective survey without retrieving data from an electronic or paper hospital database, whereby possible more variables could have been scored. Therefore, checking for bias and correcting for confounding factors was not possible. Furthermore, recall bias may be present in this survey. We requested to report on pain scores for a relatively long period after surgery, and since pain scoring then relies on the retrieval from memory, such scores become unreliable. This may have led to over- or underestimation of pain scores (but also of the use of analgesics). Still, our survey also detected a proportion of patients with present pain, which evidently is a more reliable estimation. Also, we did not gather information on the preoperative drug dosage and, therefore, the changes in the analgesic usage, which would be informative to link to pain perception and development. This must be performed in future studies.

## Conclusions

We observed that postoperative pain, particularly abdominal pain, may persist in a subset of patients undergoing bariatric surgery. In patients with pain prior to surgery, especially joint pain diminished postoperatively. New incidences of chronic pain were observed in patients with no history of any pain prior to their bariatric surgery. We contend that there is a need to achieve a consensus regarding postoperative pain management in patients after bariatric surgery.

## Supplementary information

Below is the link to the electronic supplementary material.

Supplementary file1 (136 KB)
