# Correction to: Cross-Sectional E-survey on the Incidence of Pre- and Postoperative Chronic Pain in Bariatric Surgery

**DOI:** 10.1007/s11695-022-06395-0

**Published:** 2022-12-03

**Authors:** Bart Torensma, Mohamed Hany, Marije J. S. Bakker, Monique van Velzen, Bas A. in ’t Veld, Albert Dahan, Dingeman J. Swank

**Affiliations:** 1 Department of Anaesthesiology, Leiden University Medical Center (LUMC), Albinusdreef 2, 2333 ZA Leiden, the Netherlands; 2 Medical Research Institute, Alexandria University, Alexandria, Egypt; 3 Haaglanden Medical Center, the Hauge, the Netherlands; 4 Dutch Obesity Clinic West, the Hauge, the Netherlands

**Correction to: Obesity Surgery**

**https://doi.org/10.1007/s11695-022-06354-9**

The correct name of the second author is Mohamed Hany. The correct affiliations for author Bart Torensma are 1 and 3.

