# Supplementary material for: Cross-sectional E-survey on the Incidence of Pre- and Postoperative Chronic Pain in Bariatric Surgery
Source: Obes Surg. 2022 Nov 8;33(1):204–10. doi: 10.1007/s11695-022-06354-9 (PMC9834162; doi:10.1007/s11695-022-06354-9)
Supplement: Supplementary file 1 — Supplementary file1 (136 KB) [file 11695_2022_6354_MOESM1_ESM.pdf]

Surveys of Dutch chronic pain study in obese patients - version 14.21

Printed on 07-10-2022 04:35:35 by Bart Torensma

Survey 'Survey chronisch pijn na de operatie bij NOK west'

Survey chronisch pijn na de operatie bij NOK west - Algemene gegevens.

Number	Question	Answers
	Rechts boven kunt u de knoppen op de Nederlandse taal zetten	
1.1	Wilt u deelnemen aan dit onderzoek?	◯ Ja<br>◯ Nee
	U kunt nu de volgende vragen invullen.	
	Einde onderzoek. Druk rechtsonder op volgende tot u de vragenlijst kan afsluiten	
	De volgende vragen hebben betrekking op uw algemene gegevens.	
1.1.4	***If 'Wilt u deelnemen aan dit onderzoek?' is equal to 'Ja' answer this question:***<br>Wat is uw geboortedatum?	☐ ☐ ☐ *(dd-mm-yyyy)*
1.1.5	***If 'Wilt u deelnemen aan dit onderzoek?' is equal to 'Ja' answer this question:***<br>Wat is uw geslacht?	◯ Man<br>◯ Vrouw
1.1.6	***If 'Wilt u deelnemen aan dit onderzoek?' is equal to 'Ja' answer this question:***<br>Welk type operatie heeft u gehad bij de NOK?	◯ Sleeve<br>◯ Gastric bypass
1.1.7	***If 'Wilt u deelnemen aan dit onderzoek?' is equal to 'Ja' answer this question:***<br>Wanneer heeft deze operatie plaatsgevonden?	☐ *(yyyy)*
1.1.8	***If 'Wilt u deelnemen aan dit onderzoek?' is equal to 'Ja' answer this question:***<br>Heeft u hierna nog een heroperatie gehad?	◯ Ja<br>◯ Nee

1.1.8.1	***If 'Heeft u hierna nog een heroperatie gehad?' is equal to 'Ja' answer this question:***<br>Wat was de reden voor de heroperatie?	◯ Perforatie<br>◯ Hernia<br>◯ Bloeding<br>◯ Passage probleem<br>◯ Pijnklachten
1.1.9	***If 'Wilt u deelnemen aan dit onderzoek?' is equal to 'Ja' answer this question:***<br>Wat is uw lengte in cm?	[ ] lengte in cm.
1.1.10	***If 'Wilt u deelnemen aan dit onderzoek?' is equal to 'Ja' answer this question:***<br>Wat was uw gewicht voor u geopereerd werd?	[ ] gewicht in kilogram.
1.1.11	***If 'Wilt u deelnemen aan dit onderzoek?' is equal to 'Ja' answer this question:***<br>Wat is uw gewicht op dit moment?	[ ] gewicht in kilogram.
	Klik rechts onder op "VOLGENDE"	

Survey chronisch pijn na de operatie bij NOK west - Voor uw operatie.

Number	Question	Answers
	De volgende vragen hebben betrekking op uw pijn voordat u geopereerd werd. Met langdurige pijn wordt bedoeld: langer dan 3 maanden.	
2.1	***If 'Wilt u deelnemen aan dit onderzoek?' is equal to 'Ja' answer this question:***<br>Had u last van langdurige pijn (meer dan 3 maanden) voordat u geopereerd was?	◯ Ja<br>◯ Nee
2.1.1	***If 'Had u last van langdurige pijn (meer dan 3 maanden) voordat u geopereerd was?' is equal to 'Ja' answer this question:***<br>Wat voor cijfer zou u deze pijn geven?	(0.00) — 10 (10.00) mate van pijn
2.1.2	***If 'Had u last van langdurige pijn (meer dan 3 maanden) voordat u geopereerd was?' is equal to 'Ja' answer this question:***<br>Hoe ervoer u deze pijn?	◯ Geen pijn<br>◯ Matige pijn<br>◯ Veel pijn
2.1.3	***If 'Had u last van langdurige pijn (meer dan 3 maanden) voordat u geopereerd was?' is equal to 'Ja' answer this question:***	☐ Hoofd<br>☐ Bovenarmen / schouders / nek

Number	Question	Answers
	Wat was de locatie van deze pijn?	☐ Handen / onderarmen<br>☐ Borst<br>☐ Buik<br>☐ Rug<br>☐ Bekken / billen / bovenbenen<br>☐ Knieën<br>☐ Onderbenen / voeten<br>☐ Geslachtsdelen
2.1.4	***If 'Had u last van langdurige pijn (meer dan 3 maanden) voordat u geopereerd was?' is equal to 'Ja' answer this question:***<br>Hoe verliep deze pijn?	○ Constant en aanwezig in dezelfde mate<br>○ Constant aanwezig met afwisselende pijnpunten<br>○ Constant aanwezig en toenemend in de loop van de dag<br>○ Constant aanwezig en afnemend in de loop van de dag<br>○ In aanvallen die geleidelijk verergeren, afwisselend met milde pijn<br>○ In aanvallen die geleidelijk verergeren, afwisselend met geen pijn<br>○ In plotselinge aanvallen, afwisselend met milde pijn<br>○ In plotselinge aanvallen, afwisselend met geen pijn<br>○ Verschillend in patroon van aanwezigheid van pijn
	Klik rechts onder op "VOLGENDE"	

Survey chronisch pijn na de operatie bij NOK west - Op de afdeling.

Number	Question	Answers
	De volgende vragen hebben betrekking op eventuele pijn op de verpleegafdeling, de plek waar u bent bijgekomen/ opgenomen na de operatie.	
3.1	***If 'Wilt u deelnemen aan dit onderzoek?' is equal to 'Ja' answer this question:***<br>Had u last van pijn op de verpleegafdeling?	○ Ja<br>○ Nee
3.1.1	***If 'Had u last van pijn op de verpleegafdeling?' is equal to 'Ja' answer this question:***<br>Wat voor cijfer zou u deze pijn geven?	(0.00) … 10 (10.00) mate van

			pijn
3.1.2	***If 'Had u last van pijn op de verpleegafdeling?' is equal to 'Ja' answer this question:*** Hoe ervoer u deze pijn?	Geen pijn<br>Matige pijn<br>Veel pijn	
	Klik rechts onder op "VOLGENDE"		

Survey chronisch pijn na de operatie bij NOK west - Toen u thuis kwam.

Number	Question	Answers	
	De volgende vragen hebben betrekking op eventuele pijn bij thuiskomst: het moment dat weer thuis kwam na de operatie.		
4.1	***If 'Wilt u deelnemen aan dit onderzoek?' is equal to 'Ja' answer this question:*** Had u last van pijn in de eerste week van thuiskomst?	Ja<br>Nee	
4.1.1	***If 'Had u last van pijn in de eerste week van thuiskomst?' is equal to 'Ja' answer this question:*** Wat voor cijfer zou u deze pijn geven?	(0.00)	10 (10.00) mate van pijn
4.1.2	***If 'Had u last van pijn in de eerste week van thuiskomst?' is equal to 'Ja' answer this question:*** Hoe ervoer u deze pijn?	Geen pijn<br>Matige pijn<br>Veel pijn	
	Klik rechts onder op "VOLGENDE"		

Survey chronisch pijn na de operatie bij NOK west - Op dit moment.

Number	Question	Answers
	De volgende vragen hebben betrekking op eventuele pijn op dit moment.	
5.1	***If 'Wilt u deelnemen aan dit onderzoek?' is equal to 'Ja' answer this question:***	Ja<br>Nee

Heeft u nu last van pijn?

5.1.1	***If 'Heeft u nu last van pijn?' is equal to 'Ja' answer this question:***<br>Wat voor cijfer geeft u deze pijn?	(1.00) (10.00) mate van pijn.
5.1.2	***If 'Heeft u nu last van pijn?' is equal to 'Ja' answer this question:***<br>Wat is de locatie van deze pijn?	☐ Hoofd<br>☐ Bovenarmen / schouders / nek<br>☐ Handen / onderarmen<br>☐ Borst<br>☐ Buik<br>☐ Rug<br>☐ Bekken / billen / bovenbenen<br>☐ Knieën<br>☐ Onderbenen / voeten<br>☐ Geslachtsdelen
5.1.3	***If 'Heeft u nu last van pijn?' is equal to 'Ja' answer this question:***<br>Wat is het beloop van deze pijn?	○ Constant en aanwezig in dezelfde mate<br>○ Constant aanwezig met afwisselende pijnpunten<br>○ Constant aanwezig en toenemend in de loop van de dag<br>○ Constant aanwezig en afnemend in de loop van de dag<br>○ In aanvallen die geleidelijk verergeren, afwisselend met milde pijn<br>○ In aanvallen die geleidelijk verergeren, afwisselend met geen pijn<br>○ In plotselinge aanvallen, afwisselend met milde pijn<br>○ In plotselinge aanvallen, afwisselend met geen pijn<br>○ Verschillend in patroon van aanwezigheid van pijn
5.1.4	***If 'Heeft u nu last van pijn?' is equal to 'Ja' answer this question:***<br>Hoe lang heeft u al last van deze pijn?	[ ] tijd in maanden.
5.1.5	***If 'Heeft u nu last van pijn?' is equal to 'Ja' answer this question:***<br>Hoe vaak heeft u per dag last van deze pijn?	[ ] pijnklachten per dag.
5.1.6	***If 'Heeft u nu last van pijn?' is equal to 'Ja' answer this question:***<br>Wanneer heeft u meer last van deze pijn?	☐ 's ochtends<br>☐ 's middags<br>☐ 's avonds<br>☐ 's nachts<br>☐ na maaltijden

		☐ bij w.c. bezoek
5.1.7	***If 'Heeft u nu last van pijn?' is equal to 'Ja' answer this question:***<br>Pijn patronen	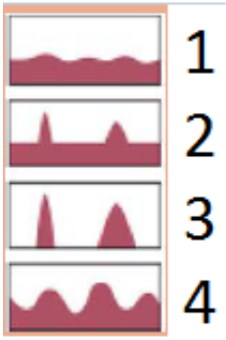
5.1.8	***If 'Heeft u nu last van pijn?' is equal to 'Ja' answer this question:***<br>Wat is het patroon van uw pijn?	◯ 1<br>◯ 2<br>◯ 3<br>◯ 4
5.1.9	***If 'Heeft u nu last van pijn?' is equal to 'Ja' answer this question:***<br>Gebruikt u medicatie tegen uw pijn?	◯ Ja<br>◯ Nee
5.1.9.1	***If 'Gebruikt u medicatie tegen uw pijn?' is equal to 'Ja' answer this question:***<br>Welke medicatie gebruikt u tegen uw pijn? (Meerdere antwoorden mogelijk)	☐ Paracetamol (Apotel, Daro, Panadol, Pinex)<br>☐ Ibuprofen (Advil, Brufen, Nurofen, Sarixell, Spidifen, Zafen)<br>☐ Diclofenac (Cataflam,, Voltaren)<br>☐ Naproxen (Aleve)<br>☐ Tramadol (Tramal, Zaldiar, Tramagetic)<br>☐ Morfine (Oramorph, Kapanol, MS Contin)<br>☐ Oxycodon (OxyContin, OxyNorm, Targinact)<br>☐ Fentanyl (Durogesic, Fentanyl Pleisters, Ionsys)<br>☐ Pregabaline (Lyrica)<br>☐ Gabapentine (Neurontin)<br>☐ Duloxetine (Cymbalta, Xeristar)<br>☐ Amitriptyline (Sarotex)<br>☐ Paroxetine (Seroxat)<br>☐ Fluoxetine (Prozac)<br>☐ Sertraline (Zoloft)<br>☐ Citalopram (Cipramil)<br>☐ Venlafaxine (Efexor)<br>☐ Lorazepam (Temesta)<br>☐ Diazepam (Diazemuls, Stesolid)<br>☐ Bromazepam<br>☐ Lormetazepam (Noctamid)<br>☐ Nitrazepam<br>☐ Etoricoxib (Arcoxia, Auxib)<br>☐ Nortriptyline (Nortrilen)

		☐ Carbamazepine (Tegretol)<br>☐ Lidocaine (Versatis)<br>☐ Capsaïcine crème<br>☐ Capsaïcine pleister (Qutenza)<br>☐ Codeïne (Paracetamol/Codeïne)<br>☐ Paracetamol/Coffeïne (Panadol)<br>☐ Acetylsalicylzuur/Paracetamol/Coffeïne (APC, Excedrin)<br>☐ Paracetamol/Propyfenazon/Coffeïne (Daro, Paradon, Sanalgin, Saridon)	
5.1.9.1.1	***If 'Welke medicatie gebruikt u tegen uw pijn? (Meerdere antwoorden mogelijk)' is equal to 'Capsaïcine crème' answer this question:***<br>Welke capsaïcine crème gebruikt u?	○ Crème (0,025%)<br>○ Crème (0,075%)	
5.1.9.1.2	***If 'Welke medicatie gebruikt u tegen uw pijn? (Meerdere antwoorden mogelijk)' is equal to 'Paracetamol (Apotel, Daro, Panadol, Pinex)' answer this question:***<br>Hoeveel mg paracetamol gebruikt u per dag?	(1000.00)	(4000.00) mg
5.1.9.1.3	***If 'Welke medicatie gebruikt u tegen uw pijn? (Meerdere antwoorden mogelijk)' is equal to 'Ibuprofen (Advil, Brufen, Nurofen, Sarixell, Spidifen, Zafen)' answer this question:***<br>Hoeveel mg ibuprofen gebruikt u per dag?	(200.00)	(1200.00) mg
5.1.9.1.4	***If 'Welke medicatie gebruikt u tegen uw pijn? (Meerdere antwoorden mogelijk)' is equal to 'Diclofenac (Cataflam,, Voltaren)' answer this question:***<br>Hoeveel mg diclofenac gebruikt u per dag?	(12.50)	(100.00) mg
5.1.9.1.5	***If 'Welke medicatie gebruikt u tegen uw pijn? (Meerdere antwoorden mogelijk)' is equal to 'Naproxen (Aleve)' answer this question:***<br>Hoeveel mg naproxen gebruikt u per dag?	(250.00)	(1500.00) mg
5.1.9.1.6	***If 'Welke medicatie gebruikt u tegen uw pijn? (Meerdere antwoorden mogelijk)' is equal to 'Tramadol (Tramal, Zaldiar, Tramagetic)' answer this question:***<br>Hoeveel mg tramadol gebruikt u per dag?	(25.00)	(200.00) mg
5.1.9.1.7	***If 'Welke medicatie gebruikt u tegen uw pijn? (Meerdere antwoorden mogelijk)' is equal to 'Morfine (Oramorph, Kapanol, MS Contin)' answer this question:***<br>Hoeveel mg morfine gebruikt u per dag?	(10.00)	(150.00) mg
5.1.9.1.8	***If 'Welke medicatie gebruikt u tegen uw pijn?***	(5.00)	(80.00) mg

	(Meerdere antwoorden mogelijk)' is equal to 'Oxycodon (OxyContin, OxyNorm, Targinact)' answer this question:<br>Hoeveel mg oxycodon gebruikt u per dag?		
5.1.9.1.9	***If 'Welke medicatie gebruikt u tegen uw pijn? (Meerdere antwoorden mogelijk)' is equal to 'Fentanyl (Durogesic, Fentanyl Pleisters, Ionsys)' answer this question:***<br>Hoeveel mcg fentanyl (pleister) gebruikt u per dag?	(25.00)	(250.00) mcg
5.1.9.1.10	***If 'Welke medicatie gebruikt u tegen uw pijn? (Meerdere antwoorden mogelijk)' is equal to 'Pregabaline (Lyrica)' answer this question:***<br>Hoeveel mg pregabaline gebruikt u per dag?	(75.00)	(300.00) mg
5.1.9.1.11	***If 'Welke medicatie gebruikt u tegen uw pijn? (Meerdere antwoorden mogelijk)' is equal to 'Gabapentine (Neurontin)' answer this question:***<br>Hoeveel mg gabapentine gebruikt u per dag?	(100.00)	(800.00) mg
5.1.9.1.12	***If 'Welke medicatie gebruikt u tegen uw pijn? (Meerdere antwoorden mogelijk)' is equal to 'Duloxetine (Cymbalta, Xeristar)' answer this question:***<br>Hoeveel mg duloxetine gebruikt u per dag?	(30.00)	(240.00) mg
5.1.9.1.13	***If 'Welke medicatie gebruikt u tegen uw pijn? (Meerdere antwoorden mogelijk)' is equal to 'Amitriptyline (Sarotex)' answer this question:***<br>Hoeveel mg amitriptyline gebruikt u per dag?	(10.00)	(100.00) mg
5.1.9.1.14	***If 'Welke medicatie gebruikt u tegen uw pijn? (Meerdere antwoorden mogelijk)' is equal to 'Paroxetine (Seroxat)' answer this question:***<br>Hoeveel mg paroxetine gebruikt u per dag?	(10.00)	(100.00) mg
5.1.9.1.15	***If 'Welke medicatie gebruikt u tegen uw pijn? (Meerdere antwoorden mogelijk)' is equal to 'Fluoxetine (Prozac)' answer this question:***<br>Hoeveel mg fluoxetine gebruikt u per dag?	(20.00)	(120.00) mg
5.1.9.1.16	***If 'Welke medicatie gebruikt u tegen uw pijn? (Meerdere antwoorden mogelijk)' is equal to 'Sertraline (Zoloft)' answer this question:***<br>Hoeveel mg sertraline gebruikt u per dag?	(25.00)	(200.00) mg
5.1.9.1.17	***If 'Welke medicatie gebruikt u tegen uw pijn? (Meerdere antwoorden mogelijk)' is equal to 'Citalopram (Cipramil)' answer this question:***<br>Hoeveel mg citalopram gebruikt u per dag?	(10.00)	(100.00) mg
5.1.9.1.18	***If 'Welke medicatie gebruikt u tegen uw pijn?***		(300.00)

	(Meerdere antwoorden mogelijk)' is equal to 'Venlafaxine (Efexor)' answer this question: Hoeveel mg venlafaxine gebruikt u per dag?	(37.50)	mg
5.1.9.1.19	***If 'Welke medicatie gebruikt u tegen uw pijn? (Meerdere antwoorden mogelijk)' is equal to 'Lorazepam (Temesta)' answer this question:*** Hoeveel mg lorazepam gebruikt u per dag?	(0.50)	(10.00) mg
5.1.9.1.20	***If 'Welke medicatie gebruikt u tegen uw pijn? (Meerdere antwoorden mogelijk)' is equal to 'Diazepam (Diazemuls, Stesolid)' answer this question:*** Hoeveel mg diazepam gebruikt u per dag?	(2.50)	(50.00) mg
5.1.9.1.21	***If 'Welke medicatie gebruikt u tegen uw pijn? (Meerdere antwoorden mogelijk)' is equal to 'Bromazepam' answer this question:*** Hoeveel mg bromazepam gebruikt u per dag?	(3.00)	(30.00) mg
5.1.9.1.22	***If 'Welke medicatie gebruikt u tegen uw pijn? (Meerdere antwoorden mogelijk)' is equal to 'Lormetazepam (Noctamid)' answer this question:*** Hoeveel mg lormetazepam gebruikt u per dag?	(1.00)	(10.00) mg
5.1.9.1.23	***If 'Welke medicatie gebruikt u tegen uw pijn? (Meerdere antwoorden mogelijk)' is equal to 'Nitrazepam' answer this question:*** Hoeveel mg nitrazepam gebruikt u per dag?	(5.00)	(40.00) mg
5.1.9.1.24	***If 'Welke medicatie gebruikt u tegen uw pijn? (Meerdere antwoorden mogelijk)' is equal to 'Etoricoxib (Arcoxia, Auxib)' answer this question:*** Hoeveel mg etoricoxib gebruikt u per dag?	(60.00)	(150.00) mg
5.1.9.1.25	***If 'Welke medicatie gebruikt u tegen uw pijn? (Meerdere antwoorden mogelijk)' is equal to 'Nortriptyline (Nortrilen)' answer this question:*** Hoeveel mg notriptyline gebruikt u per dag?	(10.00)	(100.00) mg
5.1.9.1.26	***If 'Welke medicatie gebruikt u tegen uw pijn? (Meerdere antwoorden mogelijk)' is equal to 'Carbamazepine (Tegretol)' answer this question:*** Hoeveel mg carbamazepine gebruikt u per dag?	(100.00)	(800.00) mg
5.1.9.1.27	***If 'Welke medicatie gebruikt u tegen uw pijn? (Meerdere antwoorden mogelijk)' is equal to 'Lidocaine (Versatis)' answer this question:*** Hoeveel mg lidocaine gebruikt u per dag?	(700.00)	(2100.00) mg
5.1.9.1.1.1	***If 'Welke capsaïcine crème gebruikt u?' is equal to 'Crème (0,025%)' answer this question:*** Hoeveel mg capsaïcine crème (0,025%) gebruikt u per	1 vinger	10 vingers (2.50)

	dag?	(0.25)	mg
5.1.9.1.1.2	***If 'Welke capsaïcine crème gebruikt u?' is equal to 'Crème (0,075%)' answer this question:***<br>Hoeveel mg capsaïcine crème (0,075%) gebruikt u per dag?	1 vinger (0.75)	10 vingers (7.50) mg
5.1.9.1.28	***If 'Welke medicatie gebruikt u tegen uw pijn? (Meerdere antwoorden mogelijk)' is equal to 'Capsaïcine pleister (Qutenza)' answer this question:***<br>Hoeveel mg capsaïcine pleister gebruikt u per dag?	(180.00)	(720.00) mg
5.1.9.1.29	***If 'Welke medicatie gebruikt u tegen uw pijn? (Meerdere antwoorden mogelijk)' is equal to 'Paracetamol/Coffeïne (Panadol)' answer this question:***<br>Hoeveel mg paracetamol/coffeïne gebruikt u per dag?	(550.00)	(3300.00) mg
5.1.9.1.30	***If 'Welke medicatie gebruikt u tegen uw pijn? (Meerdere antwoorden mogelijk)' is equal to 'Codeïne (Paracetamol/Codeïne)' answer this question:***<br>Hoeveel mg codeïne gebruikt u per dag?	(10.00)	(100.00) mg
5.1.9.1.31	***If 'Welke medicatie gebruikt u tegen uw pijn? (Meerdere antwoorden mogelijk)' is equal to 'Acetylsalicylzuur/Paracetamol/Coffeïne (APC, Excedrin)' answer this question:***<br>Hoeveel mg acetylsalicylzuur/paracetamol/coffeïne gebruikt u per dag?	(550.00)	(3300.00) mg
5.1.9.1.32	***If 'Welke medicatie gebruikt u tegen uw pijn? (Meerdere antwoorden mogelijk)' is equal to 'Paracetamol/Propyfenazon/Coffeïne (Daro, Paradon, Sanalgin, Saridon)' answer this question:***<br>Hoeveel mg paracetamol/propyfenazon/coffeïne gebruikt u per dag?	(450.00)	(1800.00) mg
	Klik rechts onder op "VOLGENDE"		

Survey chronisch pijn na de operatie bij NOK west - Korte Pijn Inventarisatie

Number	Question	Answers
6.1	***If 'Heeft u nu last van pijn?' is equal to 'Ja' answer this question:***<br>Tijdens ons leven hebben de meesten van ons af en toe	◯ Ja<br>◯ Nee

	pijn gehad (zoals lichte hoofdpijn, verstuikingen en kiespijn). Heeft u vandaag pijn gehad, anders dan deze allerdaagse soorten pijn?		
6.1.1	***If 'Tijdens ons leven hebben de meesten van ons af en toe pijn gehad (zoals lichte hoofdpijn, verstuikingen en kiespijn). Heeft u vandaag pijn gehad, anders dan deze allerdaagse soorten pijn?' is equal to 'Ja' answer this question:***<br>Wat is de locatie van deze pijn?	☐ Hoofd<br>☐ Bovenarmen / schouders / nek<br>☐ Handen / onderarmen<br>☐ Borst<br>☐ Buik<br>☐ Rug<br>☐ Bekken / billen / bovenbenen<br>☐ Knieën<br>☐ Onderbenen / voeten<br>☐ Geslachtsdelen	
6.1.2	***If 'Tijdens ons leven hebben de meesten van ons af en toe pijn gehad (zoals lichte hoofdpijn, verstuikingen en kiespijn). Heeft u vandaag pijn gehad, anders dan deze allerdaagse soorten pijn?' is equal to 'Ja' answer this question:***<br>Hoeveel pijn heeft u als uw pijn op zijn ERGST is?	(0.00)	10 (10.00) mate van pijn
6.1.3	***If 'Tijdens ons leven hebben de meesten van ons af en toe pijn gehad (zoals lichte hoofdpijn, verstuikingen en kiespijn). Heeft u vandaag pijn gehad, anders dan deze allerdaagse soorten pijn?' is equal to 'Ja' answer this question:***<br>Hoeveel pijn heeft u als uw pijn op zijn HET MINST is?	(0.00)	10 (10.00) mate van pijn
6.1.4	***If 'Tijdens ons leven hebben de meesten van ons af en toe pijn gehad (zoals lichte hoofdpijn, verstuikingen en kiespijn). Heeft u vandaag pijn gehad, anders dan deze allerdaagse soorten pijn?' is equal to 'Ja' answer this question:***<br>Hoeveel pijn heeft u de afgelopen 24-uur GEMIDDELD gehad?	(0.00)	10 (10.00) mate van pijn
6.1.5	***If 'Tijdens ons leven hebben de meesten van ons af en toe pijn gehad (zoals lichte hoofdpijn, verstuikingen en kiespijn). Heeft u vandaag pijn gehad, anders dan deze allerdaagse soorten pijn?' is equal to 'Ja' answer this question:***<br>Hoeveel pijn heeft u op DIT moment?	(0.00)	10 (10.00) mate van pijn
6.1.6	***If 'Tijdens ons leven hebben de meesten van ons af en toe pijn gehad (zoals lichte hoofdpijn, verstuikingen en kiespijn). Heeft u vandaag pijn gehad, anders dan deze allerdaagse soorten pijn?' is equal to 'Ja' answer this question:***<br>Hoeveel verlichting hebben pijnbehandelingen of	(0.00)	100 (100.00) mate van verlichting van pijn

	medicijnen u in de afgelopen 24 uur gegeven? Kunt u dit in een percentage tussen 0 en 100 aangeven met 0% is geen verlichting en 100% is volledige verlichting.		
	Geef het ene nummer dat het beste omschrijft hoe de pijn in de afgelopen 24-uur belemmerend heeft gewerkt op uw:		
6.1.8	***If 'Tijdens ons leven hebben de meesten van ons af en toe pijn gehad (zoals lichte hoofdpijn, verstuikingen en kiespijn). Heeft u vandaag pijn gehad, anders dan deze allerdaagse soorten pijn?' is equal to 'Ja' answer this question:***<br>ALGEMENE ACTIVITEIT: Hoeveel heeft u dit belemmerd de afgelopen 24 uur	(0.00)	10<br>(10.00) mate van belemmering
6.1.9	***If 'Tijdens ons leven hebben de meesten van ons af en toe pijn gehad (zoals lichte hoofdpijn, verstuikingen en kiespijn). Heeft u vandaag pijn gehad, anders dan deze allerdaagse soorten pijn?' is equal to 'Ja' answer this question:***<br>STEMMING: Hoeveel heeft u dit belemmerd de afgelopen 24 uur	(0.00)	10<br>(10.00) mate van belemmering
6.1.10	***If 'Tijdens ons leven hebben de meesten van ons af en toe pijn gehad (zoals lichte hoofdpijn, verstuikingen en kiespijn). Heeft u vandaag pijn gehad, anders dan deze allerdaagse soorten pijn?' is equal to 'Ja' answer this question:***<br>LOOPVERMOGEN: Hoeveel heeft u dit belemmerd de afgelopen 24 uur	(0.00)	10<br>(10.00) mate van belemmering
6.1.11	***If 'Tijdens ons leven hebben de meesten van ons af en toe pijn gehad (zoals lichte hoofdpijn, verstuikingen en kiespijn). Heeft u vandaag pijn gehad, anders dan deze allerdaagse soorten pijn?' is equal to 'Ja' answer this question:***<br>NORMALE WERKZAAMHEDEN (hieronder valt zowel werk buitenshuis als huishoudelijk werk) : Hoeveel heeft u dit belemmerd de afgelopen 24 uur	(0.00)	10<br>(10.00) mate van belemmering
6.1.12	***If 'Tijdens ons leven hebben de meesten van ons af en toe pijn gehad (zoals lichte hoofdpijn, verstuikingen en kiespijn). Heeft u vandaag pijn gehad, anders dan deze allerdaagse soorten pijn?' is equal to 'Ja' answer this question:***<br>RELATIE MET ANDERE MENSEN : Hoeveel heeft u dit belemmerd de afgelopen 24 uur	(0.00)	10<br>(10.00) mate van belemmering
6.1.13	***If 'Tijdens ons leven hebben de meesten van ons af en toe pijn gehad (zoals lichte hoofdpijn, verstuikingen en kiespijn). Heeft u vandaag pijn gehad, anders dan deze allerdaagse soorten pijn?' is equal to 'Ja' answer this***	(0.00)	10<br>(10.00) mate van belemmering

	question:<br>SLAAP: Hoeveel heeft u dit belemmerd de afgelopen 24 uur		
6.1.14	***If 'Tijdens ons leven hebben de meesten van ons af en toe pijn gehad (zoals lichte hoofdpijn, verstuikingen en kiespijn). Heeft u vandaag pijn gehad, anders dan deze allerdaagse soorten pijn?' is equal to 'Ja' answer this question:***<br>PLEZIER IN HET LEVEN: Hoeveel heeft u dit belemmerd de afgelopen 24 uur	(0.00)	10<br>(10.00) mate van belemmering
	Klik rechts onder op "VOLGENDE"		

Survey chronisch pijn na de operatie bij NOK west - Toen en nu.

Number	Question	Answers
	De volgende vragen gaan over pijn voor de operatie en pijn op dit moment.	
7.1.2	***If 'Calculatie pre- en post pijn' is equal to '1' answer this question:***<br>Was de pijn van vóór de operatie dezelfde pijn als nu?	◯ Ja<br>◯ Nee
7.1.2.1	***If 'Was de pijn van vóór de operatie dezelfde pijn als nu?' is equal to 'Ja' answer this question:***<br>Hoe is deze pijn sinds de operatie veranderd?	◯ Verminderd<br>◯ Hetzelfde<br>◯ Verergerd
	Dit onderdeel is niet bij u van toepassing. U kunt verder gaan.	
	Klik rechts onder op "VOLGENDE"	
